# Evidences of an Implemented Training Program in Consensual and Responsible Sexual Relations for People with Intellectual Disabilities

**DOI:** 10.3390/ijerph18052323

**Published:** 2021-02-26

**Authors:** Belén Gutiérrez-Bermejo, Noelia Flores, Pedro J. Amor, Cristina Jenaro

**Affiliations:** 1 PROTEDIS, Faculty of Psychology, Universidad Nacional de Educación a Distancia (UNED), 28040 Madrid, Spain; mbgutierrez@psi.uned.es; 2 INICO, Faculty of Psychology, Universidad de Salamanca, 37005 Salamanca, Spain; nrobaina@usal.es; 3 Faculty of Psychology, Universidad Nacional de Educación a Distancia (UNED), 28040 Madrid, Spain; pjamor@psi.uned.es

**Keywords:** intellectual disability, sexual behavior, interpersonal relationships, sexual health, rights, sexual abuse, sexuality education, sexual attitudes, adults, intervention

## Abstract

(1) Background: While there is a growing awareness of the rights of individuals with intellectual disabilities, very limited progress has been made in supporting these people to create and maintain intimate and personal relationships. (2) Methods: This paper reports the results from a program aimed at promoting responsible and consensual sexual relations of adults with intellectual disabilities. Of the 44 participants, 31.8% were women and 68.2% were men aged 22 to 67 years. Pre and post measurements regarding the attitudes toward sexual relations were taken, and difficulty and discrimination indexes were calculated. (3) Results: Statistically significant improvements were identified in the overall measurements, as were they for the domains of privacy, safety, and respect. The difficulty index changed from 0.67 to 0.79 in a pre-post assessment, denoting more positive attitudes. This and other results support the relevance and usefulness of the intervention program and encourage further intervention efforts.

## 1. Introduction

In the Convention on the Rights of Persons with Disabilities (CRPD) [1], sexual health, security in relationships, and a meaningful social and intimate life are recognized rights for persons with disabilities. In particular, “States Parties shall take effective and appropriate measures to eliminate discrimination against persons with disabilities in all matters relating to marriage, family, parenthood and relationships, on an equal basis with others” (article 23) [1]. While there is growing awareness of these rights, very limited progress has been made in supporting these individuals in creating and maintaining intimate and personal relationships [2]. Sexuality is a matter of equity, rights, and ethics, especially when it refers to the sexuality of people with intellectual and developmental disabilities [3]. 

When young people with intellectual disabilities begin to explore their sexuality, they face a number of challenges to access information and support. Unlike most non-disabled peers, young people with intellectual disabilities face the challenge of developing their sexuality and relationships within a very narrow and regulated environment [4]. Even though sexuality is recognized as an integral part of the adult life of any person, this part of life is denied to people with intellectual disabilities [5]. People with intellectual disabilities are seen as perpetual children who do not need to know about sex, or they are seen as sexually dangerous because they cannot adequately control their sexual instincts [6].

The opportunities people with intellectual disabilities have for expressing their sexuality are limited and controlled by others, be they caregivers or family members [7]. Thus, they are not provided with opportunities to have sexual experiences and they are not offered private environments to satisfy them [8,9,10]. Couples are not allowed to be alone, and restrictions are imposed on them [10,11] even though the CRPD states no person with disabilities, regardless of place of residence or living arrangements, shall be subjected to arbitrary or unlawful interference with his or her privacy (article 22) [1]. Staff feel uncomfortable supporting people with intellectual disabilities in developing sexual and intimate relationships [12] and their behavior is reactive rather than proactive [13,14].

Because of their circumstances, many adults with intellectual disabilities live in residences, foster homes, or with their families. These living arrangements imply the ongoing presence of an adult and a subsequent lack of privacy. These adults with intellectual disabilities do not choose who they live with, their routines, or their daily activities [6,15]. Caregivers’ attitudes directly influence the attitudes and experiences of people with intellectual disabilities, often limiting the expression of their sexual desires and their rights to live a sexual life [8,9,16]. Services for people with intellectual disabilities often lack policies that support sexual experiences in this population. Professionals who attend to them often lack training on sexuality and the sexual development of the people they serve [17]. The sexual needs of men and women with support needs are ignored; there is no concern for their need to establish friendship and partner relationships, and their sexuality and need for privacy is ignored [18,19,20]. Staff actions are focused on avoiding sexual relations, sometimes acting as “policemen” and censors of any sexual manifestation by people with intellectual disabilities.

Despite all these barriers, people with intellectual disabilities still look for ways to have sex, even in the most restrictive environments [21,22,23]. Sometimes, the behaviors of the professionals, single gender living arrangements, and single gender dressing rooms and toilets favor the development of same-sex sexual behaviors due to easier access. The sexual education people with intellectual disabilities receive is limited and rarely includes topics such as homosexuality or bisexuality. It is focused on non-reproductive heterosexual behaviors and in obtaining self-pleasure through masturbation, especially when aimed at men with intellectual disabilities [24]. Likewise, existing research focuses mainly on the prevention of sexual abuse of persons with intellectual disabilities, with limited attention being paid to other aspects of their sexuality [25]. In short, people with intellectual disabilities have very limited information on how to behave with their partners from a sexual point of view [25,26,27,28,29,30]. Moreover, as pornography is often used as a tool to learn about sex and sexual relationships [31,32,33], witnessing high-risk sexual practices in the absence of education led some young people to engage in high-risk sexual behaviors. This is particularly relevant to people with intellectual disabilities as they may internalize what is witnessed in pornography and may be more likely than the general population to act upon it [31]. Nonetheless, sexually inappropriate behaviors are considered problems in this population rather than an expression of a lack of information on sexual behaviors [5].

While the issue of sexual relations is a source of concern for professionals and parents, and actions are taken to preserve people with intellectual disabilities from abuses, pregnancy, or sexual transmitted diseases, they are being denied, at the same time, the same rights and needs that their peers without disabilities enjoy. An additional element that aggravates the issue is that there are no clear agreements or guidelines on sexual relations for this population [31], nor are there preventive or intervention programs that cover topics beyond biological and medical perspectives. Common contents in these programs are the names of female and male genitalia, the use of condoms and contraceptive methods, and information on sexually transmitted diseases [11,34]. While these programs are necessary, they are not enough. There is a complete lack of programs that go beyond these topics to include other concepts that actually serve to prevent abuse in this population [4]. Despite what we have described, we must also highlight that, despite the above, more and more organizations representing people with disabilities are claiming the right to experience sexuality as a recognized right and as a factor associated with quality of life [20]. Inspired by these principles, some very promising intervention proposals have been developed [35,36], although there are few publications reporting the outcomes and impact of their application in long-term behaviors [37]. Another example that foresees a change in this general lack of programs is the intervention aimed at improving the communication between parents and children with intellectual disabilities on sexual matters, which shows positive effects [38].

It is a challenge to find a balance between providing individuals with intellectual disabilities with the necessary protective supervision so that they are not victims of abuse, while also allowing them to enjoy their rights and freedom. The need to protect them from abuse cannot be based on the denial of a fundamental right: the right to live their sexuality. The information and training on topics related to a healthy sexuality will show them how to identify situations of abuse and how to say no to situations that they want to refuse. With adequate training and support, people with intellectual disabilities are capable of safe and constructive sexual expression and healthy relationships; this support is an essential part of aiding people with intellectual disabilities [39]. As an illustration, a review of the literature [40] shows that there is very limited information on what methods are effective in teaching sex education to people with intellectual disabilities. Regarding sexuality, the authors emphasize the need for more information about what should be taught and how it should be taught.

With regard to what needs to be taught, the literature shows that people with intellectual disabilities experience a number of issues in their sexual health. These problems are not necessarily different from people without a disability, but the degree to which they experience them is much greater. One problem is sexual abuse, which has been reported with much higher frequency in this population than in the general population [19,41,42,43]. In addition, they experience more difficulties in finding, obtaining, and maintaining desired relationships [13,19]. These disadvantages may be associated with deficits in sexual knowledge [9,44,45,46,47,48] and a lack of social and decision-making skills [49,50]. Associated with these issues is the fact that many individuals with intellectual disabilities obtain information on sex and sexual relationships via the Internet without any type of quality-based filter. This puts them at risk for abusive situations, exposing them to very limited role models on sexual behaviors and defining what and who is attractive or desirable [51]. 

Regarding comprehensive sex education, to our knowledge there are no programs that specify what it should include and how it should be taught. Some studies propose contents to be included in sex education classes [52], or they focus on social skills [50]. Other programs are aimed at the adolescent population with intellectual disabilities and their parents [36,38,53]. Other research has shown limited improvements [54]. A review of existing programs revealed that they lack specific program outcomes, do not have a theoretical basis, do not involve members of relevant groups in the development process, and lack systematic evaluation [55]. Guidelines for developing the content of these programs may rely on aspects that should be assessed when judging the sexual consent capacity of an individual with intellectual disability [56,57]. The definition of capacity and the norms for its determination are controversial [58,59]. In 1995, Ames and Samowitz [60] suggested six criteria to be used to infer that a person with intellectual disability has sexual consent capability. These criteria served as a basis in the development of our program for the promotion of healthy sex. Keep in mind that capacity is a state and not a trait. It may change over time. At any given moment, an individual with an intellectual disability may be considered as being unable to have sex because of a lack of knowledge. Subsequently, if that individual receives enough training, counseling, and exposure to various social situations, the limitation can be overcome.

The six criteria proposed [60] have served as basis to developing an updated set of criteria that emphasize desired behavior instead of non-desired behavior. (1) Voluntariness: A person must have the ability to decide voluntarily, without coercion, with whom he or she wants to have sex. In this study, we refer to this as ‘respectful of the dignity and rights of the other’. (2) Safety: Those involved in sexual behavior must be reasonably protected from physical (e.g., sexually transmitted disease) and psychological harm (e.g., unwanted separation from each other). In this study, we call this ‘safety’. (3) Non-exploitation: One should not take advantage of or use another person (e.g., someone with power or higher status) in a way that is inconsistent with willfulness. In this study, we refer to this as ‘symmetry’. (4) Non-abuse: Psychological or physical abuse should not be present in the relationship. In this study, we call this ‘mutual pleasure’. (5) Ability to say “no”: A person must be able to communicate “no” verbally or non-verbally, and withdraw from the current situation, indicating a desire to disrupt interaction. In this study, we refer to this as ‘mutual consent’. (6) Socially appropriate time and place: The person should be able to choose a socially acceptable time and place. In this study, we refer to this as “privacy”. Based on our experience, we added one more element to these criteria. (7) Affection: We understand affection as the knowledge, appreciation, esteem, and desire towards the other person, the shared experience, or toward wanting or loving that person.

In this paper, we offer the results derived from a program developed by the authors, based on these principles, as a framework for encouraging consensual and responsible sexual relationships among people with intellectual disabilities. This purpose is specified in the following objectives: (1) To analyze the sexual experience, behaviors, and attitudes towards sexual relations of adults with intellectual disabilities. (2) To implement an intervention program and obtain pre and post data on attitudes towards responsible sexual relationships. We also establish the following hypotheses: We expect to find (1) a diversity of orientations and levels of sexual experience, as occurs with the general population, and (2) pre-post improvements in the different domains of attitudes towards responsible sexual behavior.

## 2. Materials and Methods 

### 2.1. Participants

The total sample in the present study was composed of 44 participants attending an occupational center for people with intellectual disabilities in the region of Castilla y León, Spain. In these centers, occupational activities are carried out and personal and social adjustment services are received. Legally, all users of these centers were assessed for their capacities, and they met the requirements of the definition of intellectual disability. Their stay in these centers is indefinite. The level of intellectual disability in these centers ranges from mild to severe, with the most frequent assessment being moderate levels of intellectual disability. High percentages of users of these centers cannot read or write [61]. Inclusion criteria in the program were voluntary participation, as well as the authorization of their parents or guardians. The degree and type of sexual experience of the participants was not an exclusion criteria, since, from our point of view, the more diverse the groups, the more they could benefit from the exposure to diverse experiences. The study was authorized by the organization committee where the study took place. 

Of the participants, 31.8% (*n* = 14) were women and 68.2% (*n* = 30) were men. The ages ranged from 22 to 67 years (*M* = 36.48, *SD* = 12.14). The participants were attending workshops in gardening (45.5%), plastic arts (11.4%), stuffing envelopes (11.4%), handicrafts (9.1%), cleaning (9.1%), manipulative skills (6.8%), maintenance (4.5%), and recycling (2.3%). 

### 2.2. Measures and Program

In the present study, an ad hoc assessment tool was developed consisting of questions with a dichotomous response format in which participants were asked to judge if each item was correct (i.e., healthy, good, adequate) or incorrect (i.e., not healthy, bad, inappropriate). Prior to these questions, a section of sociodemographic data was collected to characterize the studied population, as well as to obtain information about the degree and type of sexual experiences they have had. In developing the attitude scale, we followed the existing recommendations [62,63,64,65,66], i.e., items should be formulated as opinions to which one can more or less agree. Through the responses provided, we can infer underlying attitude. Items, therefore, should not be formulated in the form of facts or data since we did not measure knowledge. The questionnaire was developed according to the cross-sectional axes that characterize consensual and responsible sexual relationships. As pointed out previously, they relate to: (1) mutual pleasure; (2) mutual consent; (3) privacy; (4) affection; (5) symmetry; (6) safety; (7) respectful of the dignity and rights of the other. Each axis is reflected in four items. The items were submitted to eight raters to ensure clarity in their wording, category, as well as the intensity, and valence (positive or negative). Concordance between the judges’ assessments yielded a Krippendorff’s alpha = 0.83. 

In addition, the items were written to include the three components that have traditionally been distinguished in attitudes [67]: (1) knowledge, beliefs, or the cognitive component (e.g., how to use condoms); (2) feelings or predisposition to react for or against something, in this case, different sexual behaviors or the affective component (e.g., it is important to feel affection for the other person); and (3) behaviors or behavioral intentions, that is, the conative or the behavioral component of the attitude (e.g., sexual relationships should be done in a private place). All items were rated greater than 3.2 out of 5 points in terms of the intensity with which the different domains were measured. The suggestions provided by the judges allowed us to develop a 28-item scale. Of these, 11 items include negative or “incorrect” statements, while the remaining 17 items included positive or “correct” statements. The total score is the sum of the correct items and therefore, the range is 1 to 28, after inverting negative items’ score.

The study also involved the implementation of an intervention program called “Respect for self and for others in personal relationships: prevention of sexual abuse” [68]. The program was developed with the assistance and feedback from individuals with intellectual disabilities, their families, and professionals in the field. The program is organized into six modules that address the following topics: (1) The body, genitalia, and intimacy; (2) being a man, being a woman, identity, and desire; (3) couple relationships as an option, not as a necessity; (4) the right path in sexual relationships based on respect and affection; (5) developing skills to meet another person and to be respected; (6) sexual abuse and how to prevent it; (5) adequate uses of the Internet and dangers in personal relationships; (6) when sex is bought and sold: prostitution and differences with a non-commercial sexual relationship. Each module includes introductory questionnaires to identify the existing knowledge, as well as stereotypes, negative attitudes, misinformation, etc. In addition, each module includes four worksheets that develop each theme. The activities are diverse and include group discussions, readings, hypothetical examples, individual or paired tasks, and subsequent reporting to the group. The program includes a manual for the professional that facilitates its implementation and ensures the homogeneity in its application [68].

### 2.3. Procedure

Filling out questionnaires before and after the intervention required the use of several aids: (1) reading aloud of the items, supported by a power point presentation of the items; (2) assistants to collaborate in filling in the questionnaires for those participants who lacked reading and writing skills. The implementation of the program required the following: (1) contact with the center to present the program and offer the intervention; (2) a session with parents and guardians to present the program and inform on what was to be done and request permission for the intervention; (3) a session with potential participants to provide information about the program and invite them to participate; (4) development of the evaluation tool and program; (5) program implementation and pre- and post-treatment evaluation. 

The program was completed over two weeks, with a total duration of two weeks (10 working days), for 23 h of intervention in 1.5-h sessions in the morning, plus 1.5-h in the afternoon. This was in addition to a previous 1.5-h session to apply the pre-treatment questionnaire and another 1.5-h session to apply the post-treatment questionnaire. Participants who agreed to participate were randomly divided into two groups of 22 participants each. In order to ensure the continuity of the center’s activities, participants were selected according to the workshops they attended, so that the different units did not have to be closed due to a lack of users. Participants received the training from B.G-B or C.J., two of the authors of the present study who conducted the intervention, each with one of the groups, to guarantee the homogeneity in the application of the program. In addition, the analysis of potential differences between the two groups in the studied variables revealed a lack of significant differences (Wilks’Lambda = 0.803; *F*(2, 27) = 2.214; *p* = 0.109; *ŋ_p_^2^* = 0.197). The groups were also similar in terms of age (*F*(1,40) = 0.006; *p* = 0.941) and gender (*χ^2^* = 0.043; *df* = 1; *p* = 0.837).

### 2.4. Design and Analyses

This was a pre-experimental intervention study with a one group pretest-posttest design, in which 44 participants were selected based on the workshops they attended and expressed interest in participating. These participants were randomly assigned to intervention group 1 or 2, which were identical but took place in adjoining rooms, as their intellectual disability and intragroup diversity made it advisable to work in small groups. Initial analysis involved examining frequencies for sexual behaviors, and Chi squared tests to check for the association between gender and these sexual behaviors. The main analyses required examining the percent of participants who answered each item correctly prior to and after the training course. Before analyzing pre-post changes, it was deemed appropriate to analyze the characteristics of the items in terms of difficulty and discrimination by utilizing the difficulty index and the discrimination indices, which are widely used in tests with true–false items. Those item statistics were explored to assess the performance of the participants. In performance tests, it is assumed that the test measures an underlying ability. In attitude tests, it is assumed that the test measures the underlying attitude towards an attitudinal referent (e.g., groups of people, topics, etc.). Item difficulty for true–false items is the percentage of students who answer an item correctly. The item difficulty index ranges from 0 to 100; the higher the value, the easier the question. It also plays an important role in the ability of an item to discriminate between participants who, in our case, show positive attitudes and those who do not. The item has low discrimination if almost everyone gets it wrong or guesses, or if almost everyone gets it right. For a true–false test, the ideal difficulty is 75-85 [1]. In this context, item discrimination refers to the ability of an item to differentiate among participants on the basis of how positive their attitudes are toward responsible sex. Because the discrimination index reflects the degree to which an item and the test as a whole are measuring an attribute, values of the coefficient will tend to be lower for tests measuring a wide range of domains than for more homogeneous tests. Item discrimination indices must always be interpreted in the context of the type of test that is being analyzed. Discrimination is “good” if the index is above 0.30; “fair” if it is between 0.10 and 0.30; and “poor” if it is below 0.10 [69,70]. Analyses of the difficulty and discrimination of the items helped us identify the average and the diversity in attitudes toward consensual and responsible sexual relations. Additionally, differences among the participant groups were determined by paired sample *t*-tests and Analysis of Variance, ANOVAs, with Duncan post-hoc tests. Cohen’s d effects were estimated for pair *t*-test, which is calculated as the difference between the means of each group, all divided by the standard deviation of the data. Cohen’s d effects between 0.2 < 0.5 are considered small, between 0.5 < 0.8 are medium, and ≥ 0.8 are high [71]. Figures have been created using the ggplot package [72] in the R statistical computing environment [73]. Additional analyses were completed in SPSS and an alpha = 0.05 was set for all the analyses.

## 3. Results

### 3.1. Sexual Behaviors

Regarding the sexual behaviors responses of the participants, 50% (*n* = 22) reported having or having had sex with men; 47.7% (*n* = 21) reported having or having had sex with women; 22.7% (*n* = 10) reported having sex with men and women. In addition, 31.8% (*n* = 14) indicated that they were not having sexual intercourse at the present time, and 20.5% (*n* = 9) stated that they had never had sexual intercourse. Two men (4.6%) did not offer this information. Same-sex behaviors were reported in 4.5% cases (*n* = 2), heterosexual behaviors were reported in 47.7% cases (*n* = 21), and bisexual behaviors were reported in 22.7% cases (*n* = 10).

Disaggregating the data by gender, we found that of the 30 men, 6.7% (*n* = 2) had sex only with men, 36.7% (*n* = 11) had sex only with women, and 30% (*n* = 8) had sex with both genders. In addition, 23.3% (*n* = 7) indicated that they did not have sex at present and 20% of those (*n* = 6) indicated that they had never had sex. As for the women, 71.4% (*n* = 10) reported having sex only with men, 7.1% (*n* = 1) reported having sex with both genders, and none reported having sex only with women. In addition, 21.4% of women (*n* = 3) were not having sex nor had they ever had sex. The analysis of the possible association between the gender variable and the sexual behavior indicated that it was more likely for women than for men to have heterosexual (*p* = 0.050) relationships. 

With respect to attitudes toward responsible sexual behavior, we started by analyzing item characteristics in terms of difficulty and discrimination. Table 1 provides a summary of the main characteristics. Additional information on factor composition, valence, and items’ content is included as well. In order to determine the participants’ prior attitudes, we calculated the difficulty index, understood as the percentage of correct responses in the groups of participants with extreme scores [70,72], that is, those participants from the top 25% of scores on the scale (namely, percentile 75, which in this study implied a score 21 or higher), and those participants from the bottom 25% of scores on the scale (namely, percentile 25, which in this study implied a score of 15 or less). The difficulty index is established as the average of the proportion of correct answers to each item in the two extreme groups. Therefore, a higher difficulty index indicates an easier item. In true–false questions, the expected optimal level of difficulty is 0.75; values above of 0.90 indicate that it is a very easy item and values below 0.20 indicate that the item is very difficult and has to be checked in case the writing is confusing. 

We also computed two discrimination indices [57,58]. The first index (DC1) was computed by subtracting the percentage of correct answers of the lower group from the percentage of correct answers of the upper group. Extreme values can range from −1 to +1 and negative discrimination values, which favor the lower group, should be reviewed for being poorly worded or ambiguous, etc. The questions that discriminate the best have medium difficulty, that is, values around 0.50, as this means that half of the participants responded correctly to the item. The second discrimination index (DC2) is computed by dividing the correct answers of the upper group by the sum of the correct answers of the upper and lower group. The higher the value, the more discriminating, and a value around 1 is ideal, with values equal to or greater than 0.20 being acceptable. This index tells us to what extent a question contributes to distinguishimg those who know more (or have more positive attitudes) from those who know less (or have less positive attitudes), regardless of the difficulty or ease of the question. [57]. 

Thus, Table 1 shows how the great majority of items are located in average levels of difficulty and only items 18 and 23 can be considered very easy. As for discrimination, the vast majority exceeded 0.20 in the first discrimination index and all of them exceed that value in the second discrimination index.

### 3.2. Pre-Post Changes

The difficulty index for the whole test, calculated as the ratio between the average score and the total number of items (18.74/28 = 0.67), indicated that the correct answer mean is 67%. The total discrimination index for the test, calculated as the difference between the highest and lowest score obtained, divided by the total number of items ((27-4)/28 = 0.82), supports the discriminative power of the test. Based on the responses provided by participants in the pretest, the analysis indicated significant intragroup differences (*t* = 17.26; *df* = 30; *p* < 0.001). In addition, we performed an analysis of variance using the grouping variable (i.e., lowest, intermediate, and highest groups) as a factor, and the differences were statistically significant (*F*_(2,28)_ = 74.12; *p* < 0.001). A post hoc test revealed that the three groups differed significantly from each other; the lower group scores averaged 11.11(*SD* = 3.62) correct questions, the intermediate group averaged 19.46 (*SD* = 2.26) questions, and the upper group averaged 25.33 (*SD* = 1.00). Additional analysis, including the gender variable in a 2 × 3 two-factor analysis of variance design, revealed that differences in the total of the questionnaire were significant between the three groups (*F* = 43.69; *p* < 0.001; *ŋ_p_^2^* = 0.778), but the differences were not significant for gender (*F* = 2.89, *p* = 0.060; *ŋ_p_^2^* = 0.135) or the interaction group x gender (*F* = 2.28; *p* = 0.123; *ŋ_p_^2^* = 0.154). Thus, it is possible to say that regardless of gender, the highest group (i.e., 75th percentile) differs significantly from the other two groups. Table 2 shows the results for each factor of the questionnaire, obtained by the three groups. Note that statistically significant scores were obtained in all factors except for symmetry. The post hoc analyses (Duncan) showed that the lowest group differed significantly from the other two groups.

After the intervention, the questionnaire was applied again, obtaining the results shown in Table 3. It can be seen how, globally considered, the percentage of correct answers increased in almost all of the items. Post-treatment, the difficulty index was 0.79 (= 22.1389/28), which suggests that the items were easier post-treatment for the group. The discrimination index was 0.50 ((27-13)/28), reducing the distance between participants in terms of their knowledge of the subject. These differences were not enough to reduce the intragroup differences, which remained significant (*t* = 36.16; *df* = 35; *p* < 0.001). Specifically, the three groups continue to differ significantly from each other (*F*_(2,27)_ = 39.268; *p* = 0.015) and the lowest group averaged 21.13 (SD = 1.55) correct questions, whereas the intermediate group averaged 22 (SD = 3.79) correct questions, and the upper group averaged 25.11 (SD = 1.76) correct questions. Bivariate analysis showed that there were only significant differences in ‘affection’ (*F*_(2.27)_ = 12.17; *p* <0.001) and ‘respectful’ (*F*_(2,27)_ = 8.50; *p* = 0.001). Post-hoc analysis revealed that all three groups differ significantly in “affection” and that the highest group obtained significantly higher scores in “respectful” than the remaining groups.

**Figure 1:**
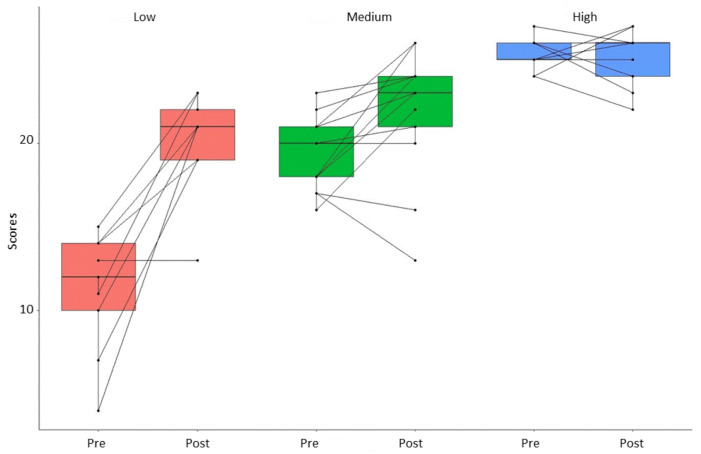
Plot of the pre-post total of within-participant pairing of data points for the three subgroups.

The total pre-post changes were significant (*t =* 4.03; *df* = 29; *p* < 0.001; *d* = 0.74), as were the following factors: Privacy (*t =* 4.37; *df* = 29; *p* < 0.001; *d* = 0.80), safety (*t =* 3.01; *df* = 29; *p* = 0.005 *d* = 0.55), and respectfulness (*t =* 2.45; *df* = 29; *p* < 0.020; *d* = 0.45). Improvements were especially high (see Table 4) for those who started the intervention program with the lowest knowledge, whereas there was a ceiling effect with the group who started with the highest knowledge; they did not change significantly. In Figure 1, the boxplot displays the changes pre-post obtained by each participant, as well as for each of the subgroups. An ascending pattern for all of the subgroups can be noted with the low group showing more intra-group progress. The high group showed the above mentioned ceiling effect. It also can be noted that post-treatment scores were different among the three subgroups.

**Figure 2:**
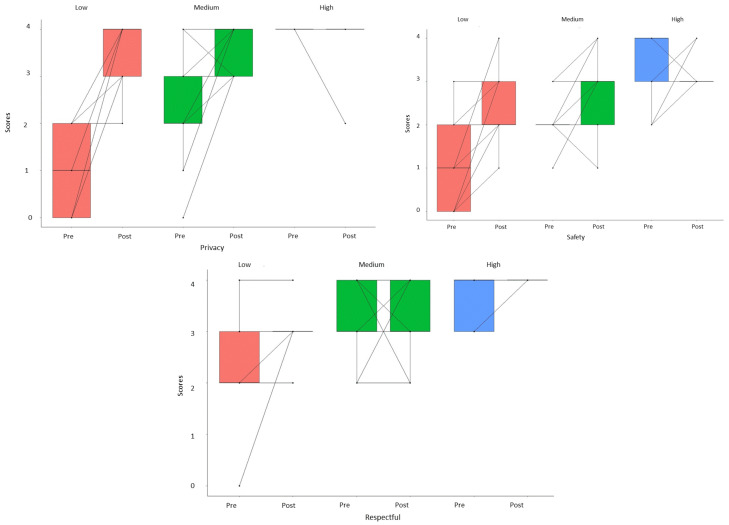
Pre-post average scores obtained by the three subgroups in the domains of privacy, safety, and respectfulness.

Figure 2 displays the pre-post scores for each of participant and subgroups in the domains where significant differences were found. Note that the low group experienced significant improvements. In fact, significant differences were obtained in privacy (*t* = 7.22, *df* = 7, *p* < 0.001), safety (*t* = 3.27, *d f*= 7, *p* = 0.01), and respectfulness (*t* = 2.55, *df* = 7, *p* = 0.04). The medium group obtained significant improvements in privacy (*t* = 3.81, *df* = 12, *p* < 0.001) and safety (*t* = 2.74, *df* = 12, *p* = 0.02), but not for respectfulness (*t* = 0.27, *df* = 12, *p* = 0.79). The high group did not obtain significant improvements in privacy (*t* = 1.00, *df* = 8, *p* < 0.35) or safety (*t* = 0.29, *df* = 8, *p* = 0.78), but the group significantly improved in respectfulness (*t* = 2.53, *df* = 8, *p* = 0.04).

## 4. Discussion

In this empirical study, we offer evidence demonstrating the usefulness of a brief intervention program to improve the knowledge and attitudes toward consensual and responsible sexual relationships in people with disabilities. This intervention leaves aside personal beliefs or values towards sexual behaviors and their diversity, which we believe is basic to promote empowerment and self-determination [74] as opposed to indoctrination towards what is “good or right” instead of “bad or wrong” in sexual behaviors.

From this point of view, the program maintained full respect for the various sexual orientations, desires, and preferences, as well as a means to satisfy them. In this study, we highlighted cross-sectional axes that must be present in a consent and responsible sexual relationship. Thus, any responsible sexual relationship should be based on mutual pleasure and consent, should take place in a context of privacy or intimacy, and should imply affection and symmetry. Further, any sexual relationship should also be safe and respectful to the dignity and rights of the other. If these cross-sectional axes are present, all sexual orientations and identities, as well as gender identities, and sexual patterns, including commercial transactions, provided that the individual with intellectual disability freely chooses to employ the services of sex workers, are considered responsible sexual relationships. We recognize that this last aspect is especially controversial, as the existing literature points out [75,76,77,78,79]. 

Returning to the results, several findings are relevant. First, the higher percentage of same sex behaviors among the male participants, in contrast to the general male population, is noteworthy [80,81,82]. What is also notable is the high percentage of people who have never had sex. These elements are, in our opinion, related. The greater access by men to sex and the greater overprotection of women could explain the high frequency of sex between men. It could be an issue of accessibility rather than one of sexual orientation. Alternative explanations could be related to the fact that the possibilities to show a variety of sexual expressions depend on the surroundings, attitudes, and behaviors toward them, and overprotective attitudes can make this fact invisible [69]. In either case, these results should be complemented with in-depth studies with additional techniques such as focus groups or qualitative interviews [69]. On the other hand, despite the traditional consideration of the “asexuality” of people with intellectual disabilities [6], the data supports the need to work on these issues since it is clear that sexual relationships among adults with intellectual disabilities are anything but anecdotal.

Concerning the results obtained in the pretest, intragroup variety is broad, although medium to high knowledge of respectful sexual behaviors generally predominates. Nonetheless, such knowledge has a wide margin for improvement that justifies interventions such as the one in this study. In addition, intragroup differences reflect a significantly higher lack of knowledge in the low performance group in the majority of factors, regardless of gender. These results highlight the urgency of intervening with a population at risk of implementing unhealthy sexual behaviors. 

The program has shown its effectiveness in improving the various components of responsible attitudes toward sexuality. While these improvements are not enough to eliminate the disadvantaged situation of the lowest performance group, they do contribute to substantially reducing the initial large difference. These improvements are compounded by the fact that the group globally improves in the pre-post scores in three of the seven factors. These results are encouraging although they will need to be replicated with a larger sample. Additional improvements are related to the time of application of the program, which should be extended. 

Before concluding, we wish to consider some shortcomings that should be addressed in further studies. First, the sample is scarce, and the analysis should be replicated with larger samples before results can be generalized. The sample size has prevented the utilization of more complex factorial designs as well. Second, as a pre-experimental design was utilized, no control or comparison group was included. Consequently, the obtained changes could be the result of other features unrelated to the treatment. We should say, though, that length of the intervention and its contents makes it difficult to attribute the changes to variables such as the maturation of the participants or the exposure to learning experiences in this regard. However, future studies with comparison or waiting list groups, and with a clear commitment to intervene with those in a latter phase, are highly recommended. Third, the questionnaire measures attitudes and as such the cognitive, affective and conative components of sexual behavior. Further studies should focus on measuring actual behavioral change. As we know, actual behavioral change is key in any program of changing attitudes and is also the most difficult to achieve [83]. Despite all of these shortcomings, we believe that the present study offers very encouraging results that support the relevance, timeliness, need, and usefulness of the intervention program.

## 5. Conclusions

This article offers evidence of the improvements obtained by adults with intellectual disabilities after participating in a program focused on training in sexual aspects from a broad perspective (rights, health, self-determination, interpersonal relationships, risks, abuse). The program showed its effectiveness in improving the various components of responsible attitudes toward sexuality, i.e., knowledge, feelings, and behavioral intentions. Further interventions should focus on measuring actual behavioral change.

## Figures and Tables

**Table 1:** Factor composition, valence, item content and difficulty and discrimination indices.

Number	Factor	Valence	Items	CDI	DC1	DC2
01	2	+	Both want to have sex at that moment	0.41	0.32	0.58
02	4	+	You like the other person	0.29	0.75	0.82
03	7	+	You are valued as a person	0.67	0.33	0.60
04	7	+	You do no physical harm	0.18	0.17	0.60
05	6	+	A condom is used	0.24	0.75	0.82
06	4	+	You know the other person beforehand (not unknown)	0.22	0.56	0.73
07	5	-	You do it with people new to the center	0.56	−0.22	0.43
08	4	+	You feel affection or something similar for the other person	0.06	0.50	0.78
09	3	-	You do it in your workplace, school, or similar.	0.29	0.75	0.82
10	7	-	You believe the other person is your property	0.67	0.33	0.60
11	1	+	You both like it	0.22	0.78	0.82
12	4	+	You sexually desire the other person	0.18	0.64	0.80
13	1	+	You do it because you want to and not out of obligation	0.33	0.67	0.75
14	6	-	Have sexually transmitted diseases	0.06	0.56	0.78
15	1	+	You care that the other person also enjoys the sex	0.29	0.75	0.82
16	2	+	The other person wants to have sex at that time	0.22	0.78	0.82
17	3	+	You do it in a private place	0.33	0.67	0.78
18	5	-	You do it with a teacher, monitor, professor or boss.	0.89	0.11	0.53
19	7	-	You humiliate, despise, mistreat or laugh at the other person	0.11	0.67	0.80
20	3	-	You record or photograph it	0.22	0.78	0.82
21	1	+	It is enjoyable for both of you	0.33	0.67	0.75
22	5	-	There is a great difference in age or they are a minor	0.22	0.11	0.57
23	5	-	One pays and the other receives money or other gifts in return	0.89	0.11	0.53
24	2	-	You do it even if the other person does not want to	0.44	0.56	0.69
25	2	+	You do it as far as the other person wants to go	0.56	0.44	0.64
26	6	+	You have sex with only one partner	0.56	0.44	0.64
27	3	-	You have sex in public places	0.22	0.78	0.82
28	6	+	You use other contraceptive methods	0.22	0.33	0.71

CDI = corrected difficulty index; DC1 = discrimination index 1; DC2 = discrimination index 2. Valence -: the items includes a negative statement. Valence +: The item includes a positive statement.

**Table 2:** Descriptive statistics and significance of differences (ANOVA) in the pretest scores between the high, medium, and low groups.

Variable	Group	*N*	M	SD	SE	*F*	*p*	*η* *^2^* *_p_*
Mutual pleasure						37.509	0.000	0.728
	Low	9	1.11	1.17	0.39			
	Medium	13	3.69	0.75	0.21			
	High	9	4.00	0.00	0.00			
Mutual consent						10.767	0.000	0.435
	Low	9	1.78	1.30	0.43			
	Medium	13	3.23	0.93	0.26			
	High	9	3.78	0.44	0.15			
Privacy						24.631	0.000	0.638
	Low	9	1.00	1.00	0.33			
	Medium	13	2.38	1.12	0.31			
	High	9	4.00	0.00	0.00			
Affection						11.063	0.000	0.441
	Low	9	1.00	1.41	0.47			
	Medium	13	2.38	1.26	0.35			
	High	9	3.56	0.53	0.18			
Symmetry						1.183	0.321	0.078
	Low	9	3.00	0.71	0.24			
	Medium	13	2.62	0.96	0.27			
	High	9	3.11	0.60	0.20			
Safety						13.404	0.000	0.489
	Low	9	1.22	1.09	.36			
	Medium	13	2.00	0.71	0.20			
	High	9	3.33	0.87	0.29			
Respectful						7.000	0.003	0.333
	Low	9	2.00	1.32	0.44			
	Medium	13	3.15	0.80	0.22			
	High	9	3.56	0.53	0.18			

N = group size; M = Mean; SD = Standard deviation; SE = Standard Error; *F* = Fisher’s F; *p* = Probability; *η*^2^_p_ = partial eta squared.

**Table 3:** Percentage of correct pre-post responses for the different questionnaire items.

Item	Pre	Post	Item	Pre	Post
it01	73.3	77.8	it15	80.0	86.1
it02	66.7	63.9	it16	67.7	58.3
it03	90.3	77.8	it17	71.0	86.1
it04	70.0	77.8	it18	87.1	94.4
it05	40.0	77.8	it19	41.9	94.4
it06	48.4	77.8	it20	45.2	91.7
it07	77.4	94.4	it21	77.4	86.1
it08	60.0	83.3	it22	58.1	58.1
it09	43.3	88.9	it23	83.3	83.3
it10	22.6	83.3	it24	25.8	25.8
it11	74.2	80.6	it25	83.9	69.4
it12	63.3	58.3	it26	83.9	80.6
it13	74.2	86.1	it27	35.5	94.4
it14	48.4	77.8	it28	41.8	97.2

**Table 4:** Pre-post participant differences grouped by group membership (Student’s T test for paired samples).

Groups		Pre	Post	*T*	*df*	*p*	*d*
**Low (*N* = 8)**				−7.933	7	<0.001	1.89
	M	10.88	21.13				
	SD	3.80	1.55				
	SE	1.34	0.55d				
**Medium (*N* = 13)**				−2.747	12	0.018	0.76
	M	19.46	22.00				
	SD	2.26	3.79				
	SE	0.63	1.05				
**High (*N* = 9)**				0.336	8	0.746	0.11
	M	25.33	25.11				
	SD	1.00	1.76				
	SE	0.33	0.59				

M = mean; SD = standard deviation; SE = standard error; t = Student’s T test; df = degrees of freedom; *p* = probability; d = Cohen’s d.

## Data Availability

Data will be provided upon request.
